# Heritable genome editing with CRISPR/Cas9 induces anosmia in a crop pest moth

**DOI:** 10.1038/srep29620

**Published:** 2016-07-12

**Authors:** Fotini A. Koutroumpa, Christelle Monsempes, Marie-Christine François, Anne de Cian, Corinne Royer, Jean-Paul Concordet, Emmanuelle Jacquin-Joly

**Affiliations:** 1 INRA, UMR iEES-Paris, route de Saint-Cyr, 78026 Versailles Cedex, France; 2 CNRS UMR 7196, INSERM U1154, Museum National d’Histoire Naturelle, Paris, France; 3 INSA-Lyon, Villeurbanne F-69621, France; 4 INRA, UMR203 BF2I, Biologie Fonctionnelle Insecte et Interaction, F-69621, France

## Abstract

Lepidoptera suffer critical lack of genetic tools and heritable genome edition has been achieved only in a few model species. Here we demonstrate that the CRISPR/Cas9 system is highly efficient for genome editing in a non-model crop pest Lepidoptera, the noctuid moth *Spodoptera littoralis*. We knocked-out the olfactory receptor co-receptor *Orco* gene to investigate its function in Lepidoptera olfaction. We find that 89.6% of the injected individuals carried *Orco* mutations, 70% of which transmitted them to the next generation. CRISPR/Cas9-mediated *Orco* knockout caused defects in plant odor and sex pheromone olfactory detection in homozygous individuals. Our work genetically defines Orco as an essential OR partner for both host and mate detection in Lepidoptera, and demonstrates that CRISPR/Cas9 is a simple and highly efficient genome editing technique in noctuid pests opening new routes for gene function analysis and the development of novel pest control strategies.

Lepidoptera represent more than 10% of the total described species of living organisms. They are studied in all fields of biological research, but suffer critical lack of reverse genetic tools. RNA interference (RNAi) approaches are usually inefficient in these species^1^ and heritable mutagenesis has been established in only a limited number of model species. First developed in *Bombyx mori* using a transposon approach^2^, such germ line transformation in other Lepidoptera has been mostly inefficient. Recently, new genome editing tools, such as Zinc Finger Nucleases (ZFN), Transcription Activator-Like Effector Nucleases (TALEN) and the Clustered Regularly Interspaced Short Palindromic Repeats (CRISPR)/Cas9 System^3^, open new routes to manipulate genes in a diversity of organisms. These methods use sequence-specific endonucleases to induce double-strand DNA breaks (DSBs) in a target gene. The non-homologous end-joining (NHEJ) DNA repair pathway is then attempting to repair the lesion, generating errors that eventually interrupt the open reading frame, inactivating the gene by incomplete protein translation^3^. ZFN-mediated mutagenesis has been recently reported for *B. mori*^4^ and *Danaus plexippus*^5^; TALEN has been developed in *B. mori*^6^,^7^,^8^; and CRISPR/Cas9 proved to be efficient in this species^9^,^10^ as well as in the model butterflies *Papilio xuthus*^11^ and *Danaus plexippus*^12^. The latter approach is in full expansion^13^,^14^ and represents a groundbreaking milestone for non-model species. The present study aims at extending its use in non-model Lepidoptera.

Focusing on the noctuid crop pest *Spodoptera littoralis*, a highly polyphagous pest causing important damage on cotton and vegetable crops in Europe, Asia and Africa, we used CRISPR/Cas9 to target a gene that could interrupt the chemical communication of this species, the receptor co-receptor Orco. As demonstrated for its orthologue in the fruit fly *Drosophila melanogaster*, Orco forms heterodimers with olfactory receptors (ORs)^15^,^16^ that act as odor-gated ion channels at the membrane of olfactory sensory neurons^17^,^18^,^19^. In *D. melanogaster*, the ORs are involved in host/partner odorant recognition while another family of receptors, the ionotropic receptors (IRs), are mostly involved in the recognition of fermentation products, such as acids and amines^20^,^21^. *Orco* disruption via knock-out or knock-down in Diptera models^16^,^22^,^23^ and other insect species^24^,^25^,^26^ induced impaired olfactory detection capacities. A noctuid *Orco* could rescue the olfactory abilities of a *D. melanogaster Orco* mutant^27^, but the essential nature of *Orco* in Lepidoptera has never been challenged yet. Thus, *Orco* represents a good target gene to investigate olfactory pathways in Lepidoptera and gives a unique opportunity for testing the development of genome engineering strategies in crop pest moths.

We report here successful CRISPR/Cas9 genome editing in a non-model pest crop moth. This work provides new perspectives for functional genomics in such emerging-species, and offers opportunities for new pest control strategies.

## Results

### *Orco* genomic structure

**Figure 1:**
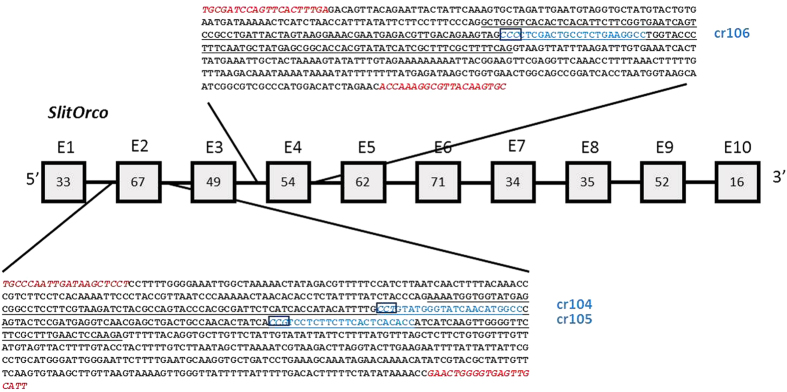
*S. littoralis Orco* (*SlitOrco*) gene structure and guide RNA positions. *SlitOrco* consists of 10 exons (grey boxes, E) and 9 introns. The number of amino acids encoded by each exon is indicated in the box. Two gRNAs, cr104 and cr105, were designed in exon 2 (E2) and one, cr106, in exon 4 (E4). Exon 2 and 4 sequences are detailed (underligned parts) showing positions of gRNAs (in blue ; PAM in italic and in box). Primers used for PCR amplification in the genotyping assay are in red italic.

The genomic sequence of the *S. littoralis Orco* gene was identified via PCR, according to the *B. mori Orco* sequence (SilkDB: http://silkworm.genomics.org.cn/) assuming that intron positions would be similar. The gene structure consisted of 10 exons and 9 introns, comparable in size and positions with its *B. mori* orthologue (Fig. 1).

### Optimization of germline transformation

As in most non-model species, *S. littoralis* embryogenesis timing is not known. We optimized early events by both selecting freshly laid eggs (within the first hour after oviposition) and injecting Cas9 protein instead of RNA, avoiding translation delay. As reasoned in Merlin *et al*.^5^, the objective was to target the beginning of the mitotic stages of the eggs, assuming that egg fertilization occurs during oviposition when sperm from the spermatheca passes through the micropyl^28^.

**Figure 2:**
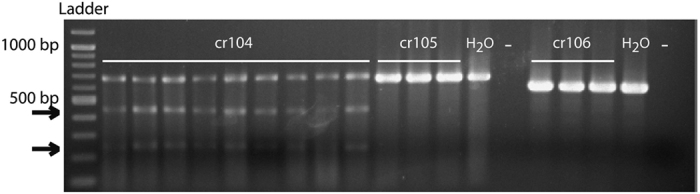
Comparison of the activity of the different guide RNAs (gRNAs). Three gRNAs (cr104, cr105, cr106) were co-injected with the Cas9 protein in different egg batches (9 batches illustrated for cr104 and 3 batches illustrated for each of the two other guides). Batches of emerging larvae were genotyped by the T7EI assay following genomic DNA extraction and PCR amplification of an *Orco* fragment (for fragment sequences and primers, see Fig. 1). DNA modification could be obtained only for cr104 and in all egg batches, as revealed by the gel pattern: the highest band corresponds to the wild type gDNA amplification and the two small bands (arrows) result from T7EI action. Water injected larvae (H_2_O) and no template (−) controls are shown for each primer combination. The ladder used is the Quick-load 2-Log DNA Ladder (0.1–10.0 kb, BioLabs).

Three gRNAs (cr104, cr105 and cr106) were designed (Fig. 1), injected in eggs and tested by genotyping pools of newly hatched larvae, to select the more efficient gRNA. PCR amplification of an *Orco* fragment encompassing the targeted sequence was followed by a T7 endonuclease I (T7EI) assay that recognized and cleaved heteroduplex DNA. By this assay, batches of larvae without any mutation revealed only one band corresponding to the wild type PCR amplified *Orco* fragment and batches of larvae containing mutations revealed two additional bands that resulted from T7EI action (Fig. 2). Out of the three gRNAs, only one was active at inducing mutations (cr104), and mutations were observed in all egg batches injected (Fig. 2). This gRNA was used further for individual studies by injection with Cas9 in 2569 eggs. In *S. littoralis*, eggs are laid in batches and percentages of injection survival (694 larvae hatched, 27%) ranged from 2.5% to 46% depending on the injected egg batch (Table 1). These percentages were similar to those observed for control eggs injected with water or just punctured (1.5% to 45%, 24% on average), suggesting that the protein/gRNA injected mix was not toxic. After individual genotyping, positive larvae were further reared until adults.

### Characterization of CRISPR/Cas9-induced mutations

**Figure 3:**
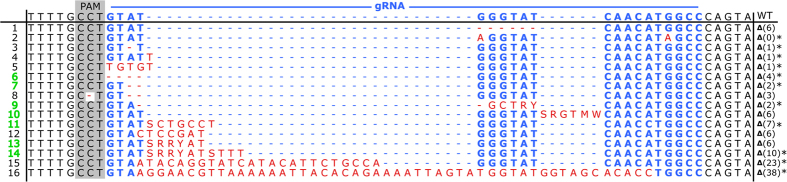
Sequences of the CRISPR/Cas9 induced mutations in the *S. littoralis Orco* gene obtained at G0. The PAM motif is highlighted as a grey box and the 20 bp cr104 guide RNA (gRNA) is shown with blue letters. The sequence modifications (Δ) are highlighted in red letters (insertions) and in red dashes (deletions). The sequences that were obtained from samples containing also sequence 1 are numbered with green numbers. The sequences with mutations that produced stop codons within the *Orco* coding sequence are designated with asterisks (*).

All data described here are summarized in Table 1. Among the larvae that emerged from cr104/Cas9-injected eggs, 58 were individually genotyped, and 89.6% (52 larvae) of those presented mismatch at the *Orco* target region (T7EI gel assay). Thirty-three were sequenced, out of which 24 harboured multiple mutations. Due to sequence superposition in chromatograms, 17 of them could not be characterised. Seven (sequences 6, 7, 9, 10, 11, 13 and 14, Fig. 3) consisted of only two or three superposed sequences and could be easily characterised by visual curation of chromatograms. Further sequencing of G1 individuals that all carried a single mutated sequence confirmed the predicted sequences. All of the seven G0 superposed sequences were carrying the same background sequence, consisting of a 6 base pair (bp) deletion (sequence 1, Fig. 3). The IUPAC code was used when the superposing bases could not be clearly identified, typically when three sequences were superposed (Fig. 3). For nine larvae (out of 33, 27%), unique mutated *Orco* sequences were detected (sequences 1–5, 8, 12, 15 and 16 in Fig. 3). Sequence 1 corresponded to the 6 bp deletion. This frequent 6 bp deletion mutation (50%: 8 out of 16 characterized mutated larvae) does not induce stop codon and has possibly no consequence on protein function. Eleven mutated sequences resulted in frameshift in the open reading frame generating a premature stop codon (sequences 2–7, 9, 11, 14, 15 and 16 in Fig. 3), and are thus expected to generate non-functional Orco proteins.

### Mutation inheritance

Ten injected individuals harbouring a unique or two different mutations (sequences 1–6, 9, 11, 15 and 16, Fig. 3) were tested for their founder (mutation transmission) capacities at the G1 generation by cross with wild-type individuals. From each cross, thirty G1 larvae randomly chosen were genotyped. Out of the ten putative founders, 8 (80%, sequences 1, 3–6, 9, 15 and 16 in Fig. 3) produced heterozygous mutant progeny at percentages ranging from 6.6 to 43.3% (Table 1), revealing mutation inheritance. Three G1 lines with mutations causing stop codons (sequences 5, 15 and 16 in Fig. 3) as well as the line with the 6 bp deletion were mated with their siblings to give second-generation mutants (G2 generation). In G2, around sixty larvae were genotyped per line. Contrary to the expected Mendelian inheritance ratio (% 25/50/25) of homozygous/heterozygous mutants/wild-types, we got an average ratio of % 5/63/32 (Table 1). Interestingly, we could not obtain any progeny after crossing homozygous mutated males and females; females laid scarce and dispersed eggs lacking scales’ protection (Supplementary Fig. S1), which dried and never hatched.

### Induced effects of *Orco* knock out

We used electroantennography (EAG) to evaluate the effect of *Orco* knock-out (KO) on the olfactory abilities of 163 G2 individuals obtained from four mutant lines (1, 5, 15 and 16 mutant lines, Fig. 3), including −/− individuals (no mutation characterized), −/+ individuals (heterozygote mutants) and +/+ individuals (homozygote mutants), all genotypes being confirmed by sequencing (Table 2). EAG was also conducted on 14 individuals from our rearing colony and whose parents did not encounter any egg injection (further designed as “wild type”). One antenna from each individual was consecutively stimulated with different plant-odorants known to be detected by *S. littoralis* antennae (E-ocimene, Z3-hexenyl-acetate, E2-hexenol, benzyl alcohol and phenyl acetaldehyde)^29^, the main sex pheromone component Z9,E11-tetradecenyl acetate (Z9,E11-14:Ac)^30^ and one acid (propionic acid) as well as their specific solvants (paraffin oil, hexane and water respectively). Plant-odorants and sex pheromones are supposed to be transduced via the OR-Orco pathway, while acids are supposed to be transduced via the IR pathway^16^,^20^,^21^,^23^,^31^. The use of these stimuli allowed us to test the effects of Orco KO on both pathways.

**Figure 4:**
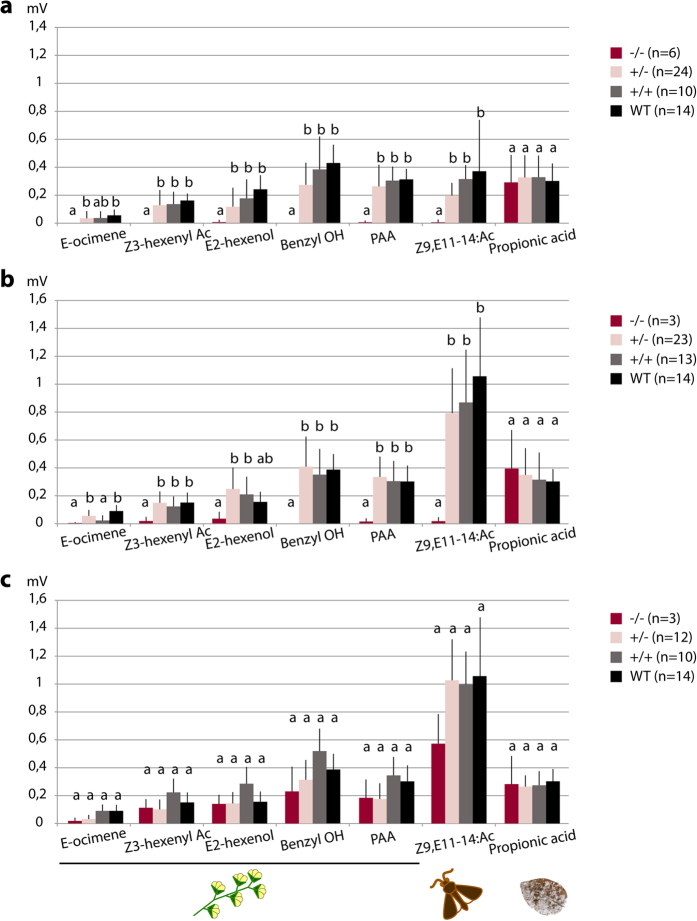
Electrophysiological impact of *Orco* KO in *S. littoralis* antennae. Electroantennogram responses (EAG, in mV ±SEM, the response to solvent was subtracted) of *S. littoralis* antennae isolated from wild-type (WT, non-injected parents) and CRISPR/Cas9 G2 individuals toward plant odorants (10 μg), the main pheromone component (Z9,E11-14:Ac, 1 μg) and propionic acid (10 μg). **(a)** Female responses: WT (black), +/+ (grey, CRISPR/Cas9 G2 without any mutation), +/− (pink, CRISPR/Cas9 G2 with heterozygous mutation) and −/− (red, CRISPR/Cas9 G2 with homozygous mutation) (sequences 5, 15 and 16 as in Fig. 3, causing a truncated Orco protein); **(b)** Male responses as in (a); **(c)** Responses from males generated from parents carrying Orco sequence 1 (2 amino acid loss compared to the Orco wild-type sequence, no stop codon produced). Different letters above each odorant response indicate significant differences (t-test; p < 0.001); a is different from b and ab is not different from either a or b. PAA: 2-phenyl acetaldehyde; Ac: acetate ; OH: alcohol; n: number of individuals tested for each genotype.

Non-mutated G2 (−/−) presented similar EAG responses as wild type adults when stimulated with plant-odorants and the main pheromone component^32^. Furthermore, we evidenced propionic acid detection in both wild type and −/− G2 males and females. The heterozygotes responded to all odorants with no statistically significant difference from the wild-types (p > 0,001, Fig. 4a,b), except for E-ocimen that induced very low EAG responses.

Whatever the sex, homozygous mutants were anosmic to the plant odorants and Z9,E11-14:Ac; the responses were significantly different from wild-type, p < 0,001, and close to zero. However, homozygous mutants responded as wild-type to the propionic acid (Fig. 4a,b). In all experiments, no difference was found between male and female, at the exception of the pheromone responses. Heterozygous and wild-type male responses to the pheromone were at least twice as high as the female responses, as previously reported^32^. The responses of individuals carrying the homozygous 6 bp mutation were not statistically different (p > 0,001) from the responses of heterozygotes and wild-types, although a weak anosmic phenotype was observed against the Z9,E11-14:Ac (Fig. 4c). This suggests that Orco was still functional for this mutation but that the two lacking amino acids (positions 61 and 62) may be important for complete functioning of the protein. Although these amino acid positions have yet never been assigned a functional role in Orco, it is known that single amino acid modification may affect Orco functioning^33^.

To test whether pheromone anosmia in *Orco* KO mutants would be responsible for the unsuccessful matings, we investigated alteration of mating performance in antennectomised wild type animals. Antennal ablation has been indeed reported to induce efficient anosmia in insects^34^,^35^. Mating capacity was not altered when the females were deprived of their antennae (100% mating and fertilized eggs, as for intact insect pairs), but was totally disrupted when we used antennectomised males (0% of mating observation and no larvae hatching).

## Discussion

Our experiments demonstrate that CRISPR technology is an effective method for generating targeted knock-outs in a non-model crop pest noctuid. We took advantage of the large number of eggs that pests usually lay in a short period, while the low number of eggs is often the major limiting parameter of induced mutagenesis/transgenesis success in other Lepidoptera. We favoured early NHEJ events by injecting gRNA/Cas9 protein within the first hour after oviposition, as close as possible to the one-nucleus-stage or the pronuclei fusion stage and surely before the blastoderm cellularisation. However, 72.7% (24 out of 33 sequenced G0 larvae) of the eggs showing effective mutation were certainly injected after the one-nucleus-stage because they carried several mutations in the *Orco* sequence. Anyhow, this strategy appears successful since genotyping and sequencing analyses performed on G1 and G2 confirmed the germline transformation, demonstrating a stable transmission of the mutations. As usually observed with CRISPR/Cas9, we obtained both deletion and insertion events. Insertions usually correspond to plasmid or genome sequence integrations. Since we did not inject any plasmid constructs, it is probable that the long fragments inserted corresponded to genome fragment, but this could not be verified since no genome is yet sequenced for *S. littoralis*.

The CRISPR/Cas9 system induced *Orco* mutations at very high efficiency (89.6%) in adults derived from injected embryos. 80% of mutated individuals transmitted mutation to their progeny with up to 43.3% germline transmission efficiency. Comparable efficiencies were obtained in *B. mori* using CRISPR/Cas9 to target the *BmBLOS2* gene^10^: 95.6% of mosaic phenotype could be obtained at G0 and germline transmission efficiency was 35.6%. Using TALEN and ZFN mutagenesis methods, 46% and 72% of G0 mutants, respectively, were obtained from *B. mori* injected embryos^4^,^6^. However, the TALEN mutagenesis gave 31% founders and a germline transmission efficiency of 61%, while the ZFNs showed very low efficiency in transmitting mutations to the G1 (9%). In monarch butterflies, 50% germline mutations has been achieved using ZFNs^5^.

In our experiment, one specific mutation (a 6 bp deletion) was found in 50% of the G0 genotyped individuals. Sequence inspection revealed that this deletion could have resulted from joining of 2 small homology stretches of sequence GTAT that flank the Cas9 cleavage site. The prevalence of this mutation may have been favoured by microhomology-mediated end-joining. This is reminiscent of studies in human cells that reported a high frequency of mutations involving stretches of sequence microhomology^36^ and suggests that when designing guide RNA for gene inactivation in *S. littoralis*, special care should be taken to avoid generating in this manner a majority of in-frame deletions as found here. As shown previously^36^, this can be done using appropriate bioinformatic tools, for example available at crispor.tefor.net or rgenome.net.

Targeted mutagenesis allowed us to knock-out the *Orco* gene in *S. littoralis*. The function of this gene in Lepidoptera has not yet been deeply investigated. As a noctuid *Orco* could rescue the detection capacities to some odorants in a *Drosophila Orco* mutant^27^, one could suggest that Orco has the same function in Lepidoptera. Here, we validate this hypothesis since the *Orco* KO homozygous mutants we obtained were not able to detect the tested host plant odorants. No effect was observed in *Orco* KO heterozygous mutants, as observed in mosquitoes^22^, suggesting that both mutated alleles are required for efficient KO. Moreover, adults were also anosmic to the main sex pheromone component. Orco coupling with the sex pheromone receptors (PRs), a well-defined subclass of Lepidoptera ORs, has been previously debated. In *B. mori*, the two *PRs* and *Orco* do not co-express in the same olfactory receptor neurons^37^, whereas co-expression would be expected if they form heterodimers. Our results suggest that lepidopteran PRs function as ORs, via interaction with Orco.

*Orco* KO homozygous mutants still detected the acid as did the wild-types. Acid sensing in Lepidoptera has not been investigated before, although they are known to express as many IRs as *D. melanogaster*, if not more^38^,^39^. Our study suggests that *S. littoralis* adults detect acids via an Orco independent pathway, probably the IR pathway.

Apart from disabling olfaction, *Orco* KO had dramatic consequences in homozygous moths. First, the homozygous ratio at G2 was lower than the expected Mendelian ratio, including in the 6 bp mutants. We suggested upper that the two lacking amino acids of these 6 bp mutants may be located in an Orco domain important for the detection of some molecules since the detection of the pheromone was slightly modified. It is possible that this Orco modification may alter the detection of some other key odorants involved in food detection, leading to an increase in larval death. Second, we could not maintain homozygotes since couples gave only few sparse eggs that never hatched. It is possible that *Orco* KO impaired sperm activity, as demonstrated in mosquitoes^40^. We cannot exclude impaired oogenesis and spermatogenesis, which were not investigated in the homozygotes, but we suspected that *Orco* KO-induced default in pheromone detection would impact mate detection. We could confirm this latter hypothesis since removing antennae in wild-type males was sufficient to impair mating, as already demonstrated in other moth species^34^,^35^. We cannot exclude that default in homozygote mating is due to off-target effects that the CRISPR/Cas9 system frequently induces^41^,^42^,^43^. Scanning the genome searching for possible additional sequences matching the gRNA was not possible since *S. littoralis* is a non-model species with no genome yet available. However, since the 6 bp deletion mutation (that did not modify the *Orco* reading frame) did not resume the olfactory responses in homozygous mutants and did not impair their reproduction, we are confident that the induced anosmia in the *Orco* KO mutants is specific.

The technological resource for noctuids described here unlocks their potential as future genetic model systems. This study appears as a proof of concept that would be potentially applicable with little adjustment to a wide variety of related species. With the development of sequencing technologies, an increased number of Lepidoptera genomes will be soon available, including those from crop pest species, thus such reverse genetic tools are highly anticipated. Modification of genomic fragments driven by pairs of double-strand breaks can be used to test the function of the targeted gene, as demonstrated here. The efficiency of this technology opens new routes since it not only allows targeting specific exons, but also putative cis-regulatory elements. This technic development may be also used for knock-in, allowing transgene replacement or even gene drive^44^, that may lead to the long term development of novel pest control technologies^45^.

## Methods

### *SlitOrco* genomic sequence

Genomic DNA was prepared from *S. littoralis* larvae using the Wizard^®^ Genomic DNA Purification Kit (Promega, Madison WI, USA) and used as template for PCRs conducted with primers designed to amplify putative intron-exon boundaries (Supplementary Table S1)

### CRISPR/Cas9 design and constructs

Three RNA guides were designed against exons 2 and 4 of the *Orco* gene (Fig. 1) using the CRISPOR gRNA design tool cripsor.tefor.net and the *SlitOrco* genomic DNA sequence as target: cr104 (5′-GGCCATGTTGATACCCATAC-3′), cr105 (5′-GGTGTGAGTGAAGAAGAGGA-3′) and cr106 (5′-GGCCTTCAGAGGCAGTCGAG-3′). Guide sequences were subcloned in DR274 (http://www.addgene.org/42250) derived vector. Plasmids were digested by DraI, purified and transcribed using Hiscribe T7 high yield transcription kit (New England Biolabs). Reactions were purified using EZNA microelute RNA clean-up kit (OMEGA Biotek). *Streptococcus pyogenes* Cas9 protein, bearing 3 nuclear localization sequences, was produced in *Escherichia coli* and purified as previously described^46^.

### Insect rearing conditions and CRISPR/Cas9 egg injections

*S. littoralis* was reared in the laboratory at 24 °C, 70% relative humidity on semi-artificial diet^47^ under light:dark 8:16 photophase. To get eggs, boxes of two to three days-old females and two males were prepared and followed up every half hour. Freshly laid egg batches were collected, scales were removed and the mix Cas9 (12.3 μM in 20 mM Hepes-NaOH pH 7.5, 150 mM KCl, 1 mM TCEP, 10% Glycerol) and gRNA (38.8 μM in water) was injected without any further dilution through the chorion using a pulled borosilicate glass capillary attached to an Eppendorf - Transjector 5246, within one hour after egg-laying. Eggs were let to develop and young larvae were reared as described above.

### Genotyping

Genomic DNA was isolated from batches of young larvae (gRNA efficiency evaluation) or from one pseudopod cut from fourth instar larvae (individual non-invasive genotyping) with the Wizard^®^ Genomic DNA Purification kit (Promega) and used as template for PCR using specific primers (for cr104 and cr105 gRNA: forward primer TGCCCAATTGATAAGCTCCT and reverse primer AATGCAACTCACCCCAGTTC; for cr106 gRNA: forward primer TGCGATCCAGTTCACTTTGA, reverse primer GCACTTGTAACGCCTTTGGT) amplifying a DNA fragment encompassing the target *Orco* sequence (Fig. 1). Mutagenic events were detected with the T7EI (New England Biolabs, Ipswich, MA USA) assay as previously described^48^ (Fig. 2). Mutated samples were sequenced at all generations (Biofidal, Vaulx-en-Velin, France). Individuals carrying KO mutations were backcrossed with wild-type adults. The G1 progeny was screened for targeted mutations as in G0, using both T7EI assay and sequencing. Male and female G1 heterozygotes carrying the same mutation were then coupled together to generate homozygous *Orco* G2 mutants. G2 were genotyped as G1 except that a wild-type *Orco* fragment was included in the PCR template to reveal homozygous mutations, and sequenced for verification.

### Phenotyping

EAG was performed on one isolated antenna from each G2 wild-type, heterozygous or homozygous adults, to evaluate the *Orco* mutation effect on the antennal capacity to detect specific odorants. Mounted between two glass electrodes containing Ringer solution^49^ and continuously humidified by charcoal-filtered airflow (70 L/h), antennae were stimulated, with a one minute interval, with 10 μg of E-ocimene, Z3-hexenyl-acetate, E2-hexenol, benzyl alcohol and 2-phenyl acetaldehyde diluted in paraffin oil, 10 μg of propionic acid in water (all purchased from Sigma-Aldrich) and 1 μg of the main sex pheromone component Z9,E11-14:Ac (Gift from Martine Lettere, Versailles, France) in hexane (Carlo Erba) (puffs of 500 ms, 10 L/h). EAG amplitudes were calculated by subtracting the solvent response. Analyses were done with Clampfit 10 software (Molecular Devices).

### Mating survey with or without antennae

Single pair matings were organized as follows: 1) male and female having both their antennae (positive control), 2) both male and female with no antennae, 3) male with no antennae and intact female and 4) intact male and female with no antennae. Six pairs of each of the three categories were kept with sugar water under the same conditions as the regular rearing and observed every two hours during three scotophases for mating. Egg laying was recorded after the 3 days and eggs were kept until larvae hatching, if any.

### Statistics

A t-test was used for statistical comparison of the EAG results.

## Additional Information

**How to cite this article**: Koutroumpa, F. A. *et al*. Heritable genome editing with CRISPR/Cas9 induces anosmia in a crop pest moth. *Sci. Rep.* **6**, 29620; doi: 10.1038/srep29620 (2016).

## Supplementary Material

Supplementary Information

## Figures and Tables

**Table 1:** Summary of the experiments from G0 to G2.

		Number	Mutated sequence number (see Fig 3)									Total	%
**Injection**	injected eggs	2569											
	hatched larvae	694											**27**
**G0**	G0 T7EI genotyped larvae	58											
	G0 mutated larvae (T7EI gel)	52											**89.6**
	G0 sequenced larvae	33											
	G0 larvae with multiple mutations (uncharacterized sequences)	17											
	G0 larvae with multiple mutations (characterized sequences)	7	6,7,9,10,11,13,14										
	G0 larvae with unique mutation (characterized sequence)	9	1–5,8,12,15,16										
	G0 sequence mutations provoking ORF frameshifts	11	2–7,9,11,14,15,16										
**Founders**	G0 mutant lines tested for founder effects (mutation transmission)	10	1–6,9,11,15,16										
	G0 founders	8	1,3–6,9,15,16										**80**
**G1 genotyping**	**Mutated sequences (as in** Fig. 3)	**1**	**2**	**3**	**4**	**5**	**6**	**9**	**11**	**15**	**16**		
	G1 genotyped larvae (T7EI)	30	30	30	30	30	30	30	30	30	30	300	
	G1 heterozygote larvae (T7EI gel)	10	10	5	6	10	5	11	11	9	13	33	
	G1 heterozygote larvae (sequencing)	10	0	3	2	7	5	11	0	6	13	57	
**Germline mutation rate**	**% heterozygote larvae (sequencing)**	**33.3**	**0**	**10**	**6.6**	**23.3**	**16.6**	**36.6**	**0**	**20**	**43.3**	**19**	
**G2**	**G2 genotyped larvae (T7E1)**	61				60				59	64	244	100
	G2 +/+ larvae (T7EI)	18				23				19	18	78	**32**
	G2 −/+ larvae (T7EI)	40				37				37	40	154	**63**
	G2 −/− larvae (T7EI, verified by sequencing) (see Table 2 for details)	3				0				3	6	12	**5**

**Table 2:** Details of G2 genotyping by *Orco* sequencing.

Sexe	mutation number	−/−	+/−	+/+
Males	1	3	12	10
Females	1	0	20	5
Males	5	0	12	2
Females	5	0	13	7
Males	15	1	9	5
Females	15	2	10	6
Males	16	2	14	8
Females	16	4	14	4

Numbers of homozygote mutants (−/−), heterozygote mutants (+/−) and wild-type individuals (+/+) obtained at the G2 generation per *Orco* mutation (see Fig. 3 for mutation numbers).
