# Supplementary material for: Heritable genome editing with CRISPR/Cas9 induces anosmia in a crop pest moth
Source: Sci Rep. 2016 Jul 12;6:29620. doi: 10.1038/srep29620 (PMC4940732; doi:10.1038/srep29620)

**Supplementary information:**

Title:

**Heritable genome editing with CRISPR/Cas9 induces anosmia in a crop pest moth**

Authors:

Fotini A. Koutroumpa^1†^, Christelle Monsempes^1†^, Marie-Christine François^1^, Anne de Cian^2^, Corinne Royer^3,4^, Jean-Paul Concordet^2^, Emmanuelle Jacquin-Joly^1^*

Author affiliations:

^1^INRA, UMR iEES-Paris, route de Saint-Cyr, 78026 Versailles Cedex, France

^2^CNRS UMR 7196, INSERM U1154, Museum National d’Histoire Naturelle, Paris, France

^3^INSA-Lyon, Villeurbanne F-69621, France

^4^INRA, UMR203 BF2I, Biologie Fonctionnelle Insecte et Interaction, F-69621, France

^†^FAK and CM contributed equally to this work

*To whom correspondance should be addressed:

Emmanuelle Jacquin-Joly, INRA, UMR iEES-Paris, route de Saint-Cyr, 78026 Versailles Cedex, France, tel. +33 1 30 83 32 12, e-mail: emmanuelle.jacquin@versailles.inra.fr

**Supplementary Table S1**

Primers designed to amplify and sequence the *S. littoralis* *Orco* gene

seqOrcog-up1 CCTGATGCCCAACATCAAGT

seqOrcog -do1 CTCGTTGACCTCATCGGAGT

seqOrcog -up2 CCATCATCAAGTTGGGGTTC

seqOrcog -do2 GCATCTGACTCCGTGAACAG

seqOrcog -up3 CGCTCACCAAGATGAGAAGA

seqOrcog -do3 CAGTCGAGGGGCTACTTCTG

seqOrcog -up4 CAGAAGTAGCCCCTCGACTG

seqOrcog -do4 TTTCACATGCGAAGATCAGC

seqOrcog -up5 TCATGGAACTCAGTGCTTCG

seqOrcog -do5 CGCAATGTCATCCCGAAATCT

seqOrcog -up6 TTGACTCCGAAGCAGGAGAT

seqOrcog -do6 GCCTGATAAGCAAGTAGAGTGAG

seqOrcog -up7 GCTGTTCCACATGTTGGTGT

seqOrcog -do7 TTCCCGAAGATACAGAAATGG

seqOrcog -up8 CTGGGACAGGTGTTCCATTT

seqOrcog -do8 AGATGCTCATGGCTTTCTGG

seqOrcog -up9 GCAGATCGTGTGCCAACAGT

seqOrcog -do9 CAGCTGTACCAACACCATGAA

**Supplementary Figure S1.** Typical egg laying patterns obtained from **a**) a wild-type mated female, **b**) a homozygous CRISPR/Cas9-induced *Orco* mutant female. Two to three-day-old males and females were put together and eggs observed 48h after. Bar: 0.5 cm.


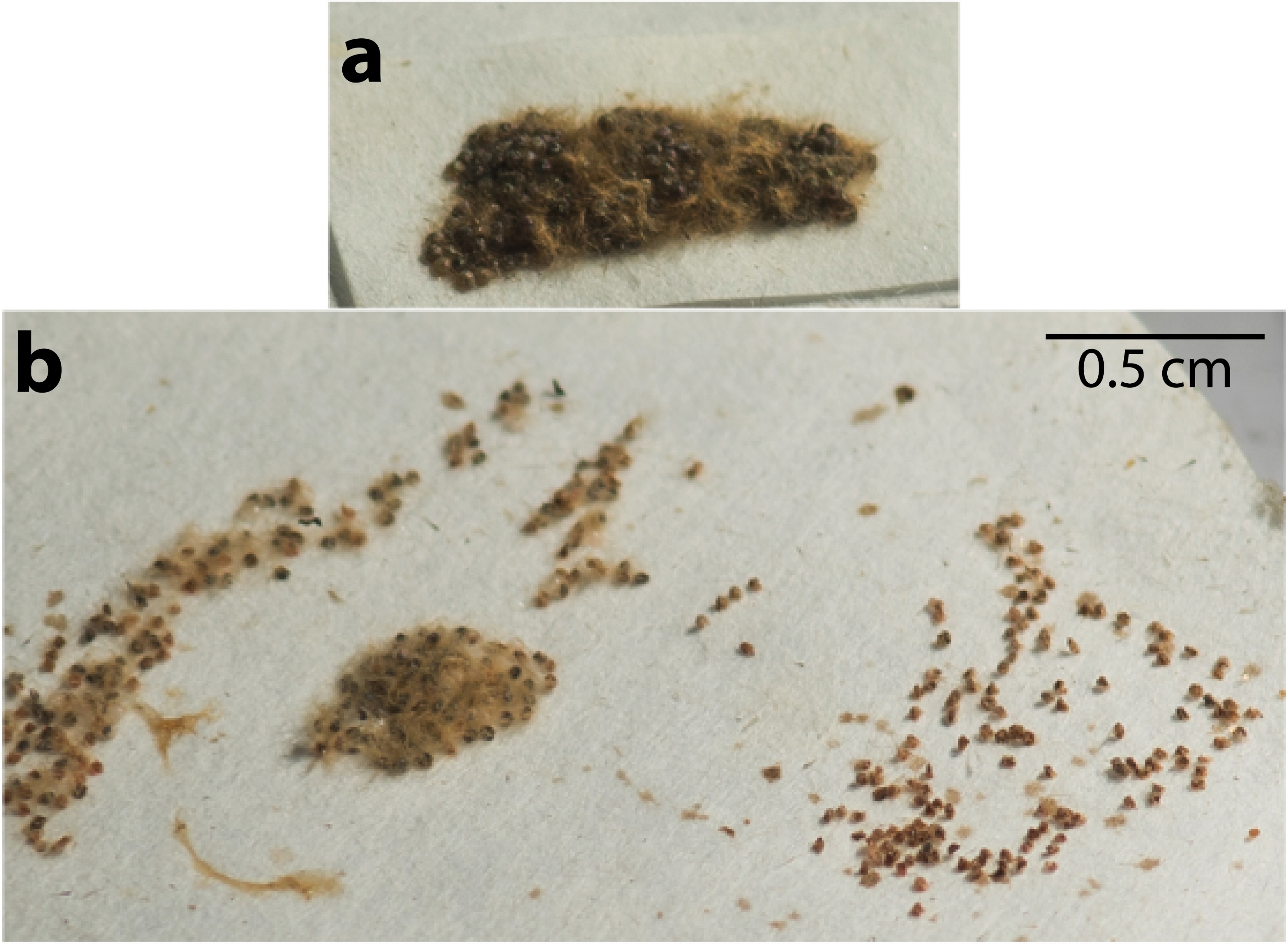

Supplement: Supplementary Information [file srep29620-s1.doc]
